# An Assessment of the Reduction of Submental Fullness With ATX-101 (Deoxycholic Acid Injection) in the Expanded Safe Zone

**DOI:** 10.7759/cureus.35286

**Published:** 2023-02-22

**Authors:** Husnain Khan, Nur Ul Ain, Dujanah S Bhatti, Junaid Khan

**Affiliations:** 1 Plastic Surgery, Holy Family Hospital, Rawalpindi, PAK; 2 Plastic and Reconstructive Surgery, Holy Family Hospital, Rawalpindi, PAK; 3 Plastic Surgery, Queen Alexandra Hospital, Portsmouth, GBR; 4 Orthopaedic Surgery, Rawalpindi Medical University, Rawalpindi, PAK

**Keywords:** facial contour, cosmetic injections, facial plastic, mesotherapy, double chin, deoxycholic acid, safe zone, complication, submental fullness, atx 101

## Abstract

Background and objective

Facial aesthetics have a huge impact on how individuals view themselves and are viewed by society. The aesthetics of the face are tremendously influenced by the shape of the chin and neck. In this study, we aimed to observe the outcomes in individuals after the use of ATX-101 (deoxycholic acid injection) in an expanded safe zone for submental fullness. To ensure optimal outcomes and reduce the risk of adverse events, appropriate patient selection is the key. ATX-101 treatment may be administered in combination with hyaluronic acid fillers, botulinum toxins, cryolipolysis, and radiofrequency treatment. This is the first study of its kind to be carried out at the national level in Pakistan.

Materials and methods

This was a quasi-experimental study conducted at the Rawalian Burn and Reconstructive Surgery Unit, Holy Family Hospital, Rawalpindi, Pakistan for a period of nine months, from 10-1-2021 to 11-10-2021. A total of 62 patients who fulfilled the inclusion criteria were enrolled. We recorded if any complications had occurred or not. Moreover, the total number of treatment sessions, the volume of injectables used, and the interval between sessions were also documented. ATX-101 package was injected into the treatment area. Due care was taken to avoid the region of the marginal mandibular nerve. After the procedure, outcomes and complications were observed.

Results

In this study, patient satisfaction was reported in 59 (95.2%) patients. After the fourth session, final improvement was observed in 59 (95.16%) patients. Tenderness was found in seven (11.3%) patients, bruising was noted in four (6.5%), edema was found in seven (11.3%), numbness was noted in one (1.6%), whereas paresis and alopecia were not found in any of the patients.

Conclusion

Our study concluded that ATX-101 is a very useful modality with fewer complication rates and is associated with significant improvement in the expanded safe zone for submental fullness.

## Introduction

The aesthetics of the face are tremendously influenced by the shape of the chin and neck. Submental fullness is a major concern for people conscious about their facial aesthetics and often prompts them to seek cosmetic treatment [1]. Excessive fat in the submental region can be a result of various factors including weight gain, genetics, and aging [2]. Submental fullness leads to an aged appearance by distorting the anterior cervicomental angle and causing skin sagging [3]. According to a survey conducted in 2017, approximately half of the participants felt that their lives were negatively impacted by submental fullness to the point of them avoiding video calls, and their pictures being taken, and some men were even prompted to grow beards to conceal this defect [4].

Previously, a surgical approach was used to treat this condition, which comprised a lower-face or neck lift. However, of late, patients’ choice of treatment options has been leaning towards nonsurgical techniques. Nonsurgical treatment options involve liposuction, low-level laser therapy, cryolipolysis, radiofrequency, injection lipolysis, etc. [5]. Among these techniques, injection lipolysis with deoxycholic acid has been gaining more and more popularity [6,7,8]. Deoxycholic acid is one of the secondary bile acids, which are metabolic byproducts of intestinal bacteria. In the human body, it is used in the emulsification of fats for absorption in the intestine. Due to these characteristics, it is used in mesotherapy injections to produce lipolysis and has been used as an alternative to surgical excision of adipose tissue. It has been approved by the Food and Drug Administration (FDA) for the reduction of submental fullness [9].

For mesotherapy of submental fullness, traditionally a one-size-fits-all approach of targeting the small central areas of submental fullness was used. This area was bordered laterally by the inferior extension of the oral commissures, superiorly by the submental crease, and inferiorly by the thyroid notch [8]. In a 2019 study, a novel expanded safe zone for treatment was introduced, which divides submental fat (SMF) compartments into six compartments, and the boundaries are extended to the submental crease superiorly, inferior neck crease inferiorly, and anterior borders of sternocleidomastoid muscle laterally. It was observed in this study that in 160 of 167 patients (95.8%), there was a reduction in submental fullness in an expanded safe zone, which led to a superior aesthetic outcome. However, they encountered complications such as edema (99.4%), numbness (97.6%), tenderness (95.8%), bruising (16.8%), alopecia (4.8%), and paresis (4.2%) [10].

There are a number of retrospective studies available on the impact of using expanded safe zone in injection lipolysis in the literature, but there is a dearth of prospective studies on this topic. Moreover, no such study has been carried out at the national level in Pakistan. In light of this, we conducted this study to validate the previous results as well as explore the outcomes at a national level.

## Materials and methods

We adopted a quasi-experimental study design (no comparison or control group) and the study was conducted in the Rawalian Burn and Reconstructive Surgery unit, Holy Family Hospital, Rawalpindi, Pakistan. The duration of the study was nine months after obtaining approval of the synopsis, i.e., 10-1-2021 to 11-10-2021.

A sample size of 62 was calculated using a 95% conﬁdence level, 4% absolute precision, and 80% power of the study, and by taking the percentage of exposed with the outcome as 95.8 [10]. The WHO calculator was used for these calculations. The sampling technique used was non-probability consecutive sampling. For sample selection, the following inclusion and exclusion criteria were used:

Inclusion criteria

Patients of either gender aged between 18-80 years presenting with submental fullness were included.

Exclusion criteria

(1) Patients not falling between the ages of 18-80 years old. (2) Other potential causes of submental convexity/fullness (e.g., excessive skin laxity, thyromegaly, submandibular ptosis, or cervical adenopathy). (3) History of use of an injectable lipolytic agent. (4) Infection in the treatment area. (5) Anatomy/landmarks or presence of scar tissue that could impact the outcome. (6) Pregnancy. (7) Use of anticoagulants.

Data collection procedure

After taking approval from the hospital's ethical committee, 62 subjects fulfilling the selection criteria were enrolled in the study from the OPD of Rawalian Burn and Reconstructive Surgery unit, Holy Family Hospital, Rawalpindi. Written informed consent was taken from all participants.

Demographic details including name, age, sex, BMI, address, and registration number were noted. The information was recorded on proforma by the researcher, which recorded the satisfaction of the surgeon, the patient, and the independent observer. It was also recorded if any complications had occurred or not. Moreover, the total number of treatment sessions, the volume of injectables used, and the interval between sessions were also recorded. This information was collected by the clinician on follow-up visits or through telephonic communication.

Treatment procedure

At the outset, expanded safe zone [10] boundaries were used to mark the submental area, as described in Table 1. The expanded safe zone comprised six zones that were assessed through both palpation by the clinician and visually to gauge submental fullness. Ten minutes before treatment, local anesthesia (lidocaine plus epinephrine) was given. Then, the 1-cm injection grid provided with the ATX-101 package (deoxycholic acid) was applied to the treatment area. This was done while carefully avoiding the area of the marginal mandibular nerve. ATX-101 (2 mg/cm^2^) was administered in 0.2-ml injections next to the grid markings and perpendicular to the surface at a depth of 6-10 mm using a 32-gauge needle. For postoperative pain management, post-injection ice (for 48 hours after treatment), and post-injection analgesia (acetaminophen) were advised.

**Table 1:** Borders of the various expanded safe zones for the injection of the ATX-101

Zones	Borders		
	Superior	Inferior	Lateral
S1	Submental crease	Thyroid notch border	Inferior extensions of oral commissures
S2	2.0 cm below the inferior border of the mandible	Thyroid notch border	Inferior extensions of oral commissure and antegonial notch
S3	2.0 cm below the inferior border of the mandible	Thyroid notch border	Inferior extension of antegonial notch and anterior border of the sternocleidomastoid muscle

Depending on the treatment goals and distribution of SMF, the total number of ATX-101 treatment sessions and the total volume of ATX-101 to be injected were tailored to each patient. According to the package insert, patients received up to six ATX-101 treatment sessions with a maximum of 10 ml per session. Patients were counseled that the usual number of treatments ranges from two to four, with approximately six weeks between sessions. On every follow-up visit, the remaining submental adiposity was assessed visually and with palpation to decide whether further treatment was needed or not.

Data analysis

All the data was entered into and analyzed using IBM SPSS Statistics version 22 (IBM Corp., Armonk, NY). The outcome variables were the reduction in submental fullness and the occurrence of various complications such as edema, numbness, tenderness, bruising, paresis, and alopecia. For quantitative variables like age and BMI, mean and standard deviation (SD) were calculated. Qualitative variables like outcome variables and gender were presented as frequency and percentage. The effective modifier was a fluctuation in the weight of the patient. Factors affecting skin laxity such as aging, racial, or genetic differences; new infections; and drugs such as steroids, antihypertensive, anti-psychotics, anti-parkinsonian agents, diuretics, anorexiants, and sedatives were controlled by stratification. Post-stratification, the chi-square test was applied. A p-value ≤0.05 was considered statistically significant.

## Results

A total of 62 patients were enrolled in the study. The mean age of the patients was 30.98 ±6.62 years (range: 19-52 years); 37 (59.68%) patients were male and 25 (40.32%) were females, with a male-to-female ratio of 1.5:1. The mean BMI of the patients was 26.96 ±3.73 kg/m^2^ (range: 19-40 kg/m^2^). 

**Figure 1:**
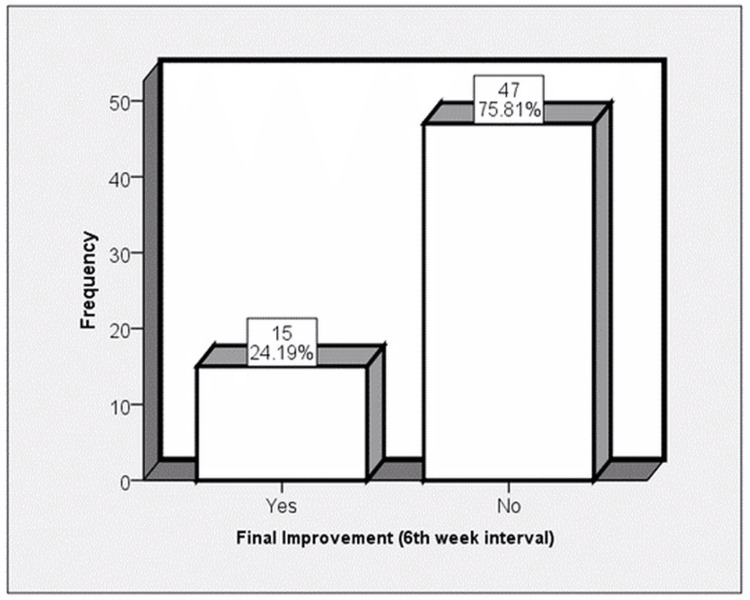
Frequency distribution of final improvement after the first session

In the first session, a 10-ml volume of injection was used in 59 (95.2%) patients and a 5-ml volume injection was used in three (4.8%) patients. Regarding satisfaction, surgeon satisfaction was found in 35 (56.5%) patients, patient satisfaction was found in 21 (33.9%) patients, and independent observer satisfaction was found in 26 (41.9%) patients (Table 2). Final improvement after the first session was noted in 15 (24.19%) patients (Figure 1).

**Table 2:** Frequency distribution of satisfaction after the first session

Satisfaction		Frequency	Percentage
Surgeon	Yes	35	56.5
	No	27	43.5
Patient	Yes	21	33.9
	No	41	66.1
Independent observer	Yes	26	41.9
	No	36	58.1

In terms of complications after the first session, tenderness was noted in four (8.5%) patients, edema was found in five (10.6%), and bruising, numbness, and alopecia were not found in any of the patients (Table 3).

**Table 3:** Frequency distribution of complications after the first session

Complications		Frequency	Percentage
Tenderness	Yes	4	8.5
	No	43	91.5
Bruising	Yes	0	0
	No	47	100.0
Edema	Yes	5	10.6
	No	42	89.4
Numbness	Yes	0	0
	No	47	100.0
Paresis	Yes	0	0
	No	47	100.0
Alopecia	Yes	0	0
	No	47	100.0

**Figure 2:**
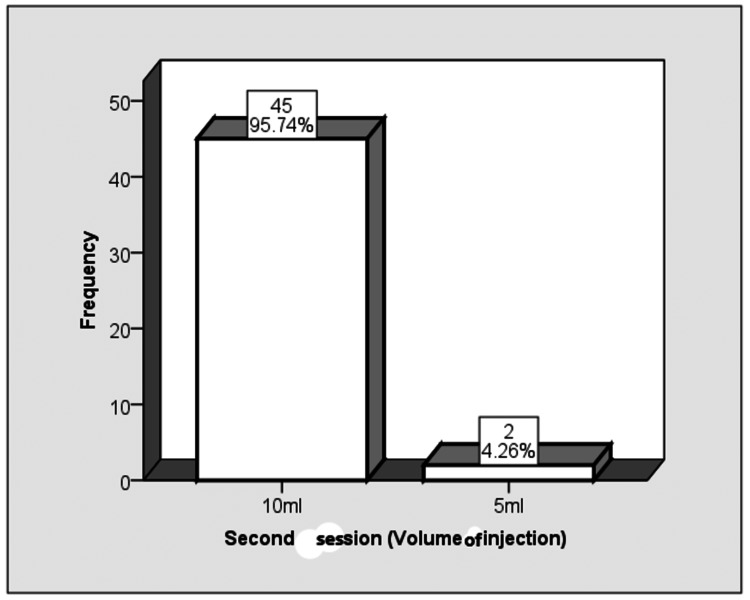
Frequency distribution of the volume of injection used (second session)

In the second session, a 10-ml volume of injection was used in 45 (95.74%) patients, and a 5-ml volume injection was used in two (4.26%) patients (Figure 2).

**Figure 3:**
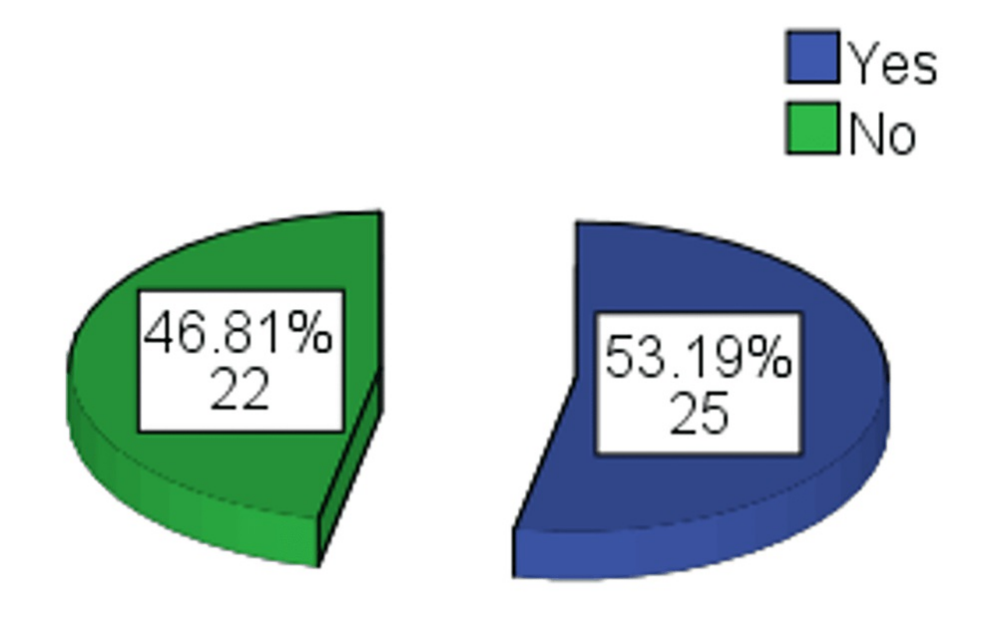
Frequency distribution of final improvement after the second session

Regarding satisfaction following the second session, surgeon satisfaction was found in 38 (80.9%) patients, patient satisfaction was found in 27 (57.4%), and independent observer satisfaction was found in 37 (78.7%) patients (Table 4). After the second session, final improvement was observed in 25 (53.19%) patients (Figure 3).

**Table 4:** Frequency distribution of satisfaction after the second session

Satisfaction (second session)		Frequency	Percentage
Surgeon	Yes	38	80.9
	No	9	19.1
Patient	Yes	27	57.4
	No	20	42.6
Independent observer	Yes	37	78.7
	No	10	21.3

In terms of complications after the second session, tenderness was noted in one (4.5%) patient, edema was found in two (9.1%), paresis was found in one (4.5%) patient, while bruising, numbness, and alopecia were not found in any of the patients (Table 5).

**Table 5:** Frequency distribution of complications after the second session

Complications (second session)		Frequency	Percentage
Tenderness	Yes	1	4.5
	No	21	95.5
Bruising	Yes	0	0
	No	22	100.0
Edema	Yes	2	9.1
	No	20	90.9
Numbness	Yes	0	0
	No	22	100.0
Paresis	Yes	1	4.5
	No	21	95.5
Alopecia	Yes	0	0
	No	22	100.0

**Figure 4:**
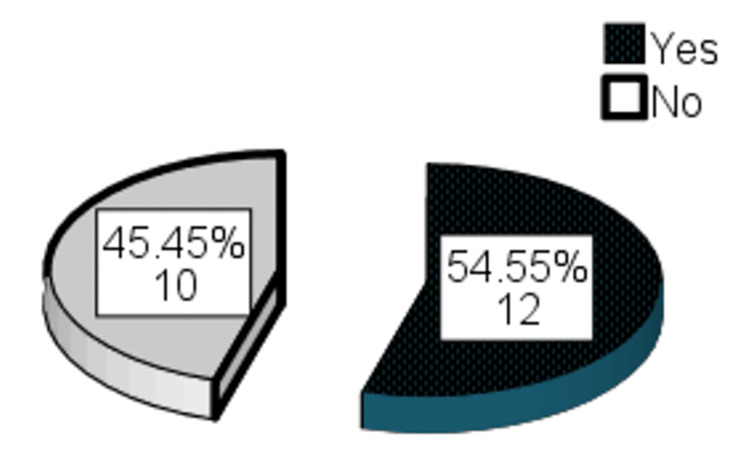
Frequency distribution of final improvement after the third session

Regarding satisfaction after the third session, surgeon satisfaction was found in 21 (95.5%) patients, patient satisfaction was found in 16 (72.7%), and independent observer satisfaction was found in 18 (81.8%) patients (Table 6). After the third session, final improvement was observed in 12 (54.55%) patients (Figure 4). No complications were noted after the third session.

**Table 6:** Frequency distribution of satisfaction after the third session

Satisfaction (third session)		Frequency	Percentage
Surgeon	Yes	21	95.5
	No	1	4.5
Patient	Yes	16	72.7
	No	6	27.3
Independent observer	Yes	18	81.8
	No	4	18.2

**Figure 5:**
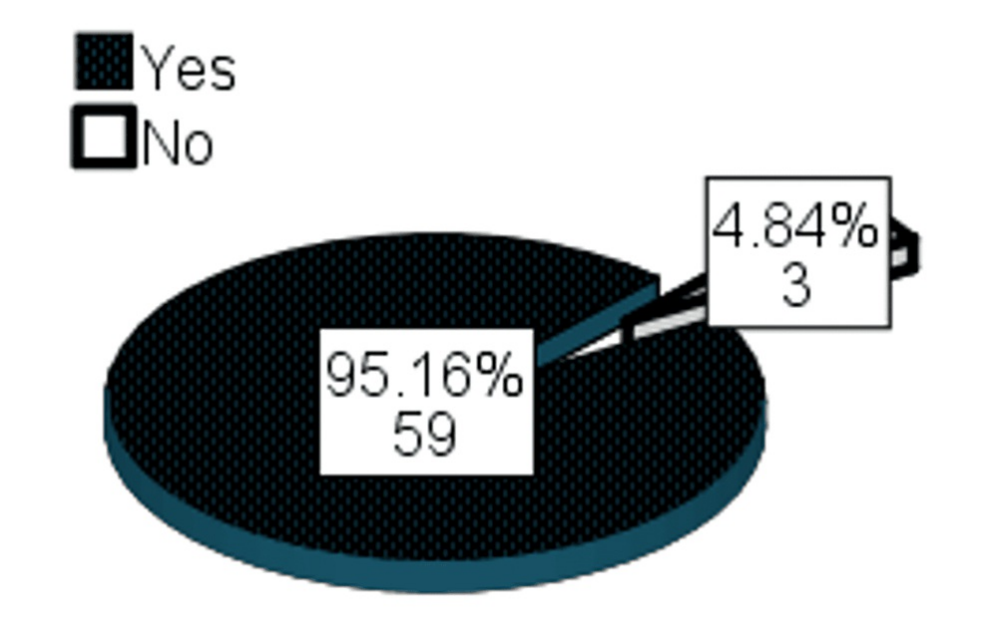
Frequency distribution of final improvement after the final session

Regarding satisfaction after the fourth session, surgeon satisfaction was found in 61 (98.4%) patients, patient satisfaction was found in 59 (95.2%), and independent observer satisfaction was found in 60 (96.77%) patients (Table 7). After the fourth session, final improvement was observed in 59 (95.16%) patients (Figure 5).

**Table 7:** Frequency distribution of satisfaction after the final session

Satisfaction (fourth session)		Frequency	Percentage
Surgeon	Yes	61	98.4
	No	1	1.6
Patient	Yes	59	95.2
	No	3	4.8
Independent observer	Yes	60	96.77
	No	2	3.2

After the final session, tenderness was found in seven (11.3%) patients, bruising was noted in four (6.5%), edema was found in seven (11.3%), numbness was noted in one (1.6%) patient, and paresis and alopecia were not found in any of the patients (Table 8).

**Table 8:** Frequency distribution of complications after the final session

Complications (fourth session)		Frequency	Percentage
Tenderness	Yes	7	11.3
	No	55	88.7
Bruising	Yes	4	6.5
	No	58	93.5
Edema	Yes	7	11.3
	No	55	88.7
Numbness	Yes	1	1.6
	No	61	98.4
Paresis	Yes	0	0
	No	62	100
Alopecia	Yes	0	0
	No	62	100

After the final session, in patients aged ≤30 years, the final improvement was noted in 35 (59.3%) patients and lack of final improvement was found in 0 (0%) patients (p=0.077); also, in patients aged ≤30 years, patient satisfaction was noted in 35 (59.3%) patients and absence of satisfaction was found in 0 (0%) patients (p=0.077) (Table 9).

**Table 9:** Comparison of final improvement and satisfaction after the final session between age groups

After final session		Age groups, years		Total	P-value
		≤30	>30		
Final improvement	Yes	35	24	59	0.077
		59.3%	40.7%	100.0%	
	No	0	3	3	
		0.0%	100.0%	100.0%	
Patients satisfaction	Yes	35	24	59	0.077
		59.3%	40.7%	100.0%	
	No	0	3	3	
		0.0%	100.0%	100.0%	

As for complications after the final session, a statistically insignificant difference was found between the age groups (p>0.05) (Table 10).

**Table 10:** Comparison of complications after the final session between age groups

Complications after the final session		Age groups, years		Total	P-value
		≤30	>30		
Tenderness	Yes	5	2	7	0.455
		71.4%	28.6%	100.0%	
	No	30	25	55	
		54.5%	45.5%	100.0%	
Bruising	Yes	2	2	4	>0.999
		50.0%	50.0%	100.0%	
	No	33	25	58	
		56.9%	43.1%	100.0%	
Edema	Yes	4	3	7	>0.999
		57.1%	42.9%	100.0%	
	No	31	24	55	
		56.4%	43.6%	100.0%	
Numbness	Yes	0	1	1	0.435
		0.0%	100.0%	100.0%	
	No	35	26	61	
		57.4%	42.6%	100.0%	

Also, a statistically insignificant difference was found between genders in terms of final improvement and satisfaction after the final session (p>0.05) (Table 11).

**Table 11:** Comparison of final improvement and satisfaction after the final session between genders

After the final session		Gender		Total	P-value
		Male	Female		
Final improvement	Yes	35	24	59	>0.999
		59.3%	40.7%	100.0%	
	No	2	1	3	
		66.7%	33.3%	100.0%	
Surgeon satisfaction	Yes	37	24	61	0.403
		60.7%	39.3%	100.0%	
	No	0	1	1	
		0.0%	100.0%	100.0%	
Patient satisfaction	Yes	35	24	59	>0.999
		59.3%	40.7%	100.0%	
	No	2	1	3	
		66.7%	33.3%	100.0%	

There was a statistically insignificant difference between genders with regard to complications after the final session (p>0.05) (Table 12).

**Table 12:** Comparison of complications after the final session between genders

Complications after the final session		Gender		Total	P-value
		Male	Female		
Tenderness	Yes	5	2	7	0.691
		71.4%	28.6%	100.0%	
	No	32	23	55	
		58.2%	41.8%	100.0%	
Bruising	Yes	2	2	4	>0.999
		50.0%	50.0%	100.0%	
	No	35	23	58	
		60.3%	39.7%	100.0%	
Edema	Yes	3	4	7	0.425
		42.9%	57.1%	100.0%	
	No	34	21	55	
		61.8%	38.2%	100.0%	
Numbness	Yes	0	1	1	0.403
		0.0%	100.0%	100.0%	
	No	37	24	61	
		60.7%	39.3%	100.0%	

A statistically insignificant difference was found regarding final improvement and satisfaction after the final session between patient groups classified according to BMI (p>0.05) (Table 13).

**Table 13:** Comparison of final improvement and satisfaction between patient groups categorized according to BMI

After final session		BMI, kg/m^2^		Total	P-value
		≤25	>25		
Final improvement	Yes	21	38	59	0.545
		35.6%	64.4%	100.0%	
	No	0	3	3	
		0.0%	100.0%	100.0%	
Surgeon satisfaction	Yes	21	40	61	>0.999
		34.4%	65.6%	100.0%	
	No	0	1	1	
		0.0%	100.0%	100.0%	
Patient satisfaction	Yes	21	38	59	0.545
		35.6%	64.4%	100.0%	
	No	0	3	3	
		0.0%	100.0%	100.0%	

A statistically insignificant difference was found regarding complications after the final session between patient groups classified according to BMI (p>0.05) (Table 14).

**Table 14:** Comparison of complications after the final session between patient groups categorized according to BMI

Complications after the final session		BMI, kg/m^2^		Total	P-value
		≤25	>25		
Tenderness	Yes	2	5	7	>0.999
		28.6%	71.4%	100.0%	
	No	19	36	55	
		34.5%	65.5%	100.0%	
Bruising	Yes	0	4	4	0.290
		0.0%	100.0%	100.0%	
	No	21	37	58	
		36.2%	63.8%	100.0%	
Edema	Yes	2	5	7	>0.999
		28.6%	71.4%	100.0%	
	No	19	36	55	
		34.5%	65.5%	100.0%	
Numbness	Yes	0	1	1	>0.999
		0.0%	100.0%	100.0%	
	No	21	40	61	
		34.4%	65.6%	100.0%	

## Discussion

Facial harmony and attractiveness are significantly impacted by the submental area. In addition, self-perception can be negatively affected by an undesirable submental profile [4,5]. Even in individuals who are not overweight, the accumulation of SMF can occur and it is usually resistant to measures taken toward weight reduction. Traditionally, treatment options to reduce submental fullness have mainly comprised surgical procedures [10-16]. In 2015, in the United States and Canada, ATX-101 was approved based on conclusions from four randomized controlled phase-3 trials, two conducted in Europe (ATX-101, n=484) and two conducted in the United States and Canada [REFINE trials (ATX-101, n=514)] [17].

In this study, after the final session, surgeon satisfaction was found in 61 (98.4%) patients, and patient satisfaction was found in 59 (95.2%) patients. After the fourth session, final improvement was observed in 59 (95.16%) patients. Tenderness was found in seven (11.3%) patients, bruising was noted in four (6.5%), edema was found in seven (11.3%), numbness was noted in one (1.6%) patient, while paresis and alopecia were not found in any of the patients.

In a recent 2019 study, a novel expanded safe zone for treatment was introduced, which divides SMF compartments into six compartments, and the boundaries are extended to the submental crease superiorly, inferior neck crease inferiorly, and anterior borders of sternocleidomastoid muscle laterally [10]. Shridharani et al. documented in their study that improvement in submental contour was achieved in 160 of 167 patients (95.8%). The majority of complications consisted of numbness, injection-site edema, and tenderness. An individualized treatment plan with ATX-101 requires careful assessment of every patient’s SMF and an understanding of submental anatomy, which allows for the treatment of areas beyond the central region of the neck without increased risk of adverse events.

Beer et al. [18] documented in their study that the efficacy and safety of ATX-101 continued over 12 months. In general, 84.9% of participants were pleased with the appearance of their faces/chins. At 12 months, 82.9% of participants remained unchanged, and 10.1% had improvement in their appearance compared to 12 weeks after the last treatment. Adverse events primarily involved the treatment area and were mostly mild to moderate in severity. During the first week after the first treatment, 33.9% of participants missed social/leisure activities, and 13.3% missed work. Following further treatment sessions, 10.0-15.7% of participants missed social/leisure activities and 2.4-6.0% missed work. During the 12-month follow-up, the positive results of ATX-101 treatment were maintained in this study. High percentages of subjects who were CR-1 or PR-1 responders at 12 weeks after the last treatment maintained the response at 12 months, consistent with follow-up data from phase 2/3 ATX-101 trials. Improvements continued during follow-up as evidenced by a >90% CR-1 response in all subjects at 12 months [18,19].

Compared with the phase-3 randomized controlled trial data for central SMF treatment, expanded safe zone treatment [10] produced a similar adverse event profile with regard to marginal mandibular nerve paresis (4.3% versus 4.8 %), which resolved over a similar period (range: 7-60 days versus 14-40 days) without sequelae [19]. Compared with the randomized controlled trials (71.7%), bruising was reported in a fewer number of patients in the study by Shridharani et al. (16.8%) [10]. However, the frequency of numbness and edema was greater in this analysis than in the randomized controlled trials (97.6% versus 66.5% and 99.4% versus 87.3%, respectively), most likely due to the increased total volume of ATX-101 administered and greater surface area treated [20].

Patients must be educated that edema and swelling are common adverse effects of ATX-101 because of its mechanism of action and in fact indicative of the progression of treatment. In the REFINE trials, edema and swelling were reported in 78.1% of patients treated with ATX-101 with a median duration of 10-11 days. Moreover, in the REFINE trials, dysphagia was reported in 1.9% of subjects, which was possibly a result of edema and swelling within the submental area [21,22].

ATX-101 is particularly beneficial in the treatment of patients with mild or moderate submental fullness. Over 90% of patients treated with ATX-101 in open-label clinical trials or the REFINE trial showed either improvement or no change in the submental fullness. However, patients with excess sub-platysmal fat or severe submental skin laxity may be better treated by alternative treatment options to address submental fullness or skin-tightening therapy along with ATX-101 administration [22,23]. Teller et al. [23] reported in their study that for the reduction of SMF, the finest clinical practices regarding the use of ATX-101 should enable physicians to boost treatment outcomes and patient experience.

One limitation of our study is that it focused on the use of ATX-101 alone for submental fullness. However, in clinical practice, ATX-101 treatment may be administered in combination with hyaluronic acid fillers, botulinum toxins, cryolipolysis, and radiofrequency treatment. For example, if the patient has extreme SMF, the CoolSculpting CoolMini applicator may be used to debulk the area, and ATX-101 can be used for subsequent fine-contouring of the submental area. In the properly selected patient, this combination treatment can result in exceptional outcomes [24].

## Conclusions

Excessive submental fullness can harmfully impact one’s perception of self-attractiveness, and increased facial adiposity is considered less healthy and undesirable. Various methods have been employed to address the issue of SMF, ranging from topical agents to injectable treatment and even surgical extraction. We have highlighted successful outcomes with an injectable option, targeting both patients' and surgeons' satisfaction with minimal complications. Although multiple sessions may be required for the treatment to be completely efficacious, we concluded that it was an acceptable option. This study concluded that ATX-101 is a very useful option with fewer complication rates and with better improvement rates in the expanded safe zone for submental fullness. However, more effective options could be explored with adjuvant treatments in combination with ATX-101 injections. We recommend the use of ATX sessions to treat SMF collection in patients not keen on surgical intervention, as it has been shown to produce significantly favorable results.

## References

[1] Shah GM, Greenberg JN, Tanzi EL, Monheit GD (2017). Noninvasive approach to treatment of submental fullness. Semin Cutan Med Surg.

[2] Raveendran SS, Anthony DJ, Ion L (2012). An anatomic basis for volumetric evaluation of the neck. Aesthet Surg J.

[3] Cheng CK (2019). High-efficiency combination treatment of submental neck fullness. Plast Reconstr Surg Glob Open.

[4] Cooke K (2018). Special feature: treating the submental area [EPUB]. Aesthet J.

[5] Pacifico MM (2019). Treating the submental area [EPUB]. Aesthet J.

[6] Shridharani SM, Behr KL (2017). ATX-101 (deoxycholic acid injection) treatment in men: insights from our clinical experience. Dermatol Surg.

[7] Deeks ED (2016). Deoxycholic acid: a review in submental fat contouring. Am J Clin Dermatol.

[8] Shamban AT (2016). Noninvasive submental fat compartment treatment. Plast Reconstr Surg Glob Open.

[9] Patel S, Kridel R (2018). Current trends in management of submental liposis: a pooled analysis and survey. JAMA Facial Plast Surg.

[10] Shridharani SM, Chandawarkar AA (2019). Novel expanded safe zone for reduction of submental fullness with ATX-101 injection. Plast Reconstr Surg.

[11] Schlessinger J, Weiss SR, Jewell M, Narurkar V, Weinkle S, Gold MH, Bazerkanian E (2013). Perceptions and practices in submental fat treatment: a survey of physicians and patients. Skinmed.

[12] Rohrich RJ, Rios JL, Smith PD, Gutowski KA (2006). Neck rejuvenation revisited. Plast Reconstr Surg.

[13] Narasimhan K, Stuzin JM, Rohrich RJ (2013). Five-step neck lift: integrating anatomy with clinical practice to optimize results. Plast Reconstr Surg.

[14] Honigman R, Castle DJ (2006). Aging and cosmetic enhancement. Clin Interv Aging.

[15] Mashkevich G, Wang J, Rawnsley J, Keller GS (2009). The utility of ultrasound in the evaluation of submental fullness in aging necks. Arch Facial Plast Surg.

[16] Gosain AK, Klein MH, Sudhakar PV, Prost RW (2005). A volumetric analysis of soft-tissue changes in the aging midface using high-resolution MRI: implications for facial rejuvenation. Plast Reconstr Surg.

[17] Humphrey S, Sykes J, Kantor J (2016). ATX-101 for reduction of submental fat: a phase III randomized controlled trial. J Am Acad Dermatol.

[18] Beer K, Weinkle SH, Cox SE, Rubin MG, Shamban A, Somogyif C (2019). ATX-101 (deoxycholic acid injection) for reduction of submental fat: results from a 12-month open-label study. J Drugs Dermatol.

[19] Ava Shamban M, Somogyif C (2019). ATX-101 (deoxycholic acid injection) for reduction of submental fat: results from a 12-month open-label study. J Drugs Dermatol.

[20] Kybella P (2023). Kybella (deoxycholic acid) injection prescribing information. Westlake Village, CA: Kythera Biopharmaceuticals Inc. Inc.

[21] Dayan SH, Schlessinger J, Beer K (2018). Efficacy and safety of ATX-101 by treatment session: pooled analysis of data from the phase 3 REFINE Trials. Aesthet Surg J.

[22] Jones DH, Carruthers J, Joseph JH (2016). REFINE-1, a multicenter, randomized, double-blind, placebo-controlled, phase 3 trial with ATX-101, an injectable drug for submental fat reduction. Dermatol Surg.

[23] Teller CF, Chiu A, Chesnut CD (2021). Best clinical practices with ATX-101 for submental fat reduction: patient-related factors and physician considerations. Plast Reconstr Surg Glob Open.

[24] Shridharani SM (2019). Real-world experience with 100 consecutive patients undergoing neck contouring with ATX-101 (deoxycholic acid): an updated report with A 2-year analysis. Dermatol Surg.

